# Traditional, complementary, and integrative medicine in a rapidly digitalizing research environment: ethics and intellectual property considerations

**DOI:** 10.1186/s12906-026-05382-7

**Published:** 2026-04-15

**Authors:** Jeremy Y. Ng

**Affiliations:** 1 Institute of General Practice and Interprofessional Care, University Hospital Tübingen, Tübingen, Germany; 2 Robert Bosch Center for Integrative Medicine and Health, Bosch Health Campus, Stuttgart, Germany; 3 Department of Health Research Methods, Evidence, and Impact, Faculty of Health Sciences, McMaster University, Hamilton, Canada; 4 School of Public Health, Faculty of Health, University of Technology Sydney, Sydney, Australia

**Keywords:** Artificial intelligence, Complementary medicine, Data sovereignty, Ethics, Intellectual property, Traditional knowledge

## Abstract

This editorial explores the intersection between artificial intelligence (AI) and traditional, complementary, and integrative medicine (TCIM), with a focus on emerging ethical and intellectual property (IP) concerns. Although AI has the potential to improve evidence synthesis, support clinical decision-making, and streamline research processes, its use in TCIM is not without challenges. Issues related to data quality, bias, and the underrepresentation of traditional knowledge systems are of particular importance. The editorial also considers how existing IP frameworks may be poorly suited to protect collectively held traditional knowledge, raising concerns about consent and benefit sharing. Thoughtful governance, greater transparency, and meaningful engagement with knowledge holders will be essential to ensure that AI applications in the context of TCIM are developed ethically.

## Introduction

Traditional, complementary, and integrative medicine (TCIM) is now firmly embedded in global health systems. The World Health Organization (WHO) has emphasized its relevance through successive global strategies and reports that highlight its widespread use, policy integration, and growing research base [1, 2]. At the same time, artificial intelligence (AI) is reshaping how medical knowledge is generated, synthesized, and applied. Two initiatives I contributed to, including the WHO’s recent technical brief “Mapping the application of artificial intelligence in traditional medicine” [3], as well as the session titled “From policy to practice – responsible AI and digital innovation in traditional, complementary, and integrative medicine”, which formed part of the broader session “Measuring progress and charting the way forward: standards, data, and responsible AI—from ancestral knowledge to action” at the Second WHO Global Summit on Traditional Medicine (https://tm-summit.org/agenda/), held in Delhi, India, in December 2025, signals that this convergence is no longer speculative but operational.

For TCIM, this technological shift is especially consequential. The field has long faced methodological heterogeneity, variable regulatory oversight, and uneven evidence quality [4, 5]. AI tools, particularly large language models, automated evidence synthesis systems, and predictive analytics, offer potential solutions. They can accelerate literature mapping, identify research gaps, support clinical decision-making, and improve access to synthesized evidence. In research settings, AI may assist with protocol development, data extraction, and quality appraisal. In clinical contexts, it may support integrative treatment planning or patient education.

Yet these benefits are not automatic. AI systems reflect the data on which they are trained and the assumptions embedded in their design. In a field characterized by pluralistic epistemologies and culturally situated practices, uncritical integration of AI risks reinforcing existing hierarchies of evidence or marginalizing knowledge systems that do not conform to biomedical norms [6]. The central question is not whether AI will shape TCIM, but how responsibly this influence will unfold.

### Ethical imperatives for AI in TCIM research and care

The ethical integration of AI into TCIM requires careful attention to research integrity, fairness, and accountability.

First, maintaining the quality and reliability of evidence is essential. AI tools trained on heterogeneous or low-quality studies may produce outputs that appear authoritative but rest on weak evidence. Automated summaries and recommendation systems must therefore be designed to communicate uncertainty and reflect methodological limitations. Without such safeguards, AI could amplify misinformation or oversimplify complex therapeutic traditions.

Second, concerns about representation and access are particularly salient in TCIM. These therapies are frequently used by populations who experience structural barriers in mainstream healthcare. If AI tools are developed primarily in high-income, English-language contexts, they may underrepresent diverse healing traditions or fail to accommodate linguistic and cultural variation. Bias in training datasets is not merely a technical limitation; it is a governance issue that shapes whose knowledge is recognized and whose is marginalized [7, 8]. TCIM encompasses knowledge systems that are historically transmitted, community-embedded, and not always found in conventional biomedical literature. Responsible integration therefore requires collaboration with traditional knowledge holders and transparency about how systems interpret and classify TCIM practices.

Finally, accountability must be clarified. When AI-informed recommendations contribute to patient harm or research error, responsibility may be diffuse. Developers, researchers, clinicians, and institutions each play a role. Given the regulatory variability that characterizes TCIM globally, clear disclosure standards and human oversight mechanisms are indispensable. AI should support, and not replace, professional judgment.

### Intellectual property and the protection of traditional knowledge

The incorporation of AI into TCIM raises profound intellectual property (IP) challenges. Traditional knowledge is often collectively held, orally transmitted, and embedded in cultural and spiritual contexts [6]. These characteristics sit uneasily within conventional IP regimes, which are typically structured around individual ownership and formal documentation.

AI development intensifies this tension. Machine learning systems are trained on large corpora that may include digitized ethnobotanical records, traditional formulations, and community-derived practices. When such data are incorporated into commercial tools without consent or benefit sharing, the process resembles digital bioprospecting [9]. The scale and speed of AI magnify this risk.

Key questions follow: Who owns outputs generated by models trained on traditional knowledge? What obligations do developers have to communities whose knowledge informs training datasets? How should benefits be shared when AI-derived products generate commercial value from culturally embedded practices?

Recent international developments, including the World Intellectual Property Organization’s treaty on IP, genetic resources, and associated traditional knowledge, reflect growing recognition of these concerns [10]. However, legal reform alone is insufficient. Ethical governance must also address data sovereignty, community participation, and transparency in dataset construction.

Indigenous data governance frameworks, such as the CARE Principles [11], emphasize collective benefit, authority to control, responsibility, and ethics. Applying such principles to AI development in TCIM would require meaningful consultation with knowledge holders, clear documentation of data provenance, and mechanisms for ongoing community engagement. These processes are complex, particularly where knowledge is shared across regions or generations. Nevertheless, failure to address them risks eroding trust and perpetuating historical patterns of extraction.

Protecting traditional knowledge in the age of AI is therefore not solely a legal exercise. It is a moral obligation grounded in cultural preservation, fairness, and scientific integrity.

### Charting a responsible path forward

AI has the potential to strengthen TCIM research and treatment. It can enhance transparency in evidence synthesis, improve access to reliable information, and support integrative clinical decision-making. For researchers, it may streamline workflows and elevate methodological rigour. For patients, it may expand access to clearer, more personalized information.

Realizing these benefits requires intentional governance. Ethical and IP considerations must be embedded at the design stage of AI systems rather than addressed retrospectively. This includes transparent reporting of data sources, explicit acknowledgment of uncertainty, safeguards against bias, and participatory models of knowledge stewardship.

Policymakers, researchers, clinicians, developers, and traditional knowledge holders must collaborate to define standards that reflect both scientific rigour and cultural respect. Capacity building in digital literacy and equitable participation in AI innovation, particularly for low- and middle-income countries, will be essential.

The future of TCIM in the digital era will be shaped not only by technological capability but by ethical clarity. AI can either reinforce inequities and accelerate knowledge extraction, or it can strengthen research integrity and global health advances. The direction chosen will depend on whether ethics and IP are treated as peripheral concerns or as structural determinants of responsible innovation.

## Data Availability

No datasets were generated or analysed during the current study.

## Abbreviations

AI: Artificial intelligence
IP: Intellectual property
TCIM: Traditional, complementary, and integrative medicine
WHO: World Health Organization
